# Pregnant Women Sharing Pregnancy-Related Information on Facebook: Web-Based Survey Study

**DOI:** 10.2196/jmir.7753

**Published:** 2018-03-22

**Authors:** Tammy Harpel

**Affiliations:** ^1^ Department of Family & Consumer Sciences Illinois State University Normal, IL United States

**Keywords:** pregnancy, social media, Facebook

## Abstract

**Background:**

Research indicates expectant and new mothers use the Internet, specifically social media, to gain information and support during the transition to parenthood. Although parents regularly share information about and photos of their child or children on Facebook, researchers have neither explored the use of Facebook to share pregnancy-related information nor investigated factors that influence such sharing.

**Objective:**

The aim of this study was to address a gap in the literature by exploring the use of Facebook by pregnant women. Specifically, the study examined the use of Facebook to share pregnancy-related information, as well as any association between prenatal attachment and the aforementioned aspects of sharing pregnancy-related information on Facebook.

**Methods:**

Pregnant women who were at least 18 years of age were recruited for participation in the study through posts and paid advertisements on Facebook and posts to professional organization listservs. Individuals interested in participating were directed to a secure Web-based survey system where they completed the consent form and the survey that focused on their current pregnancy. Participants completed the Maternal Antenatal Attachment Scale and answered questions that assessed how often they shared pregnancy-related information on Facebook, who they shared it with, why they shared it, and what they shared.

**Results:**

A total of 117 pregnant women completed the survey. Descriptive statistics indicated that the pregnancy announcement was most commonly shared (75/108, 69.4%), with most women sharing pregnancy-related information on Facebook less than monthly (52/117, 44.4%) with only family and friends (90/116, 77.6% and 91/116, 78.4%, respectively) and for the purpose of involving others or sharing the experience (62/107, 57.9%). Correlation and regression analyses showed that prenatal attachment, in general, was positively and significantly related to all aspects of sharing pregnancy-related information at the *P*<.05 level, with the exception of sharing because of expectations. Quality of attachment, which involves the positive feelings the woman has about her unborn child, was significantly associated with sharing to involve others or share the pregnancy (*t*_8,93_=2.654 *, P*=.009). In contrast, after controlling for other variables, the strength or preoccupation component of prenatal attachment was significantly associated with frequency of sharing (*t*_8,100_=2.554 *, P*=.01), number to types of information shared (*t*_8,97_=2.605 *, P*=.01), number of groups with whom shared (*t*_8,99_=3.467, *P*=.001), and sharing to get advice (χ^2^_8_=5.339 *, P*=.02).

**Conclusions:**

Pregnant women in this study used Facebook for a variety of reasons, demonstrating the use of the social media platform during pregnancy for supportive and informational purposes. Overall, the results of this study are likely to be useful to professionals who are seeking alternative methods for providing intervention, information, and support to pregnant women via social media in our technology-driven society.

## Introduction

### Social Media Use and Parents

The transition to parenthood, which begins at pregnancy and continues through the postpartum period, has been altered from decades past by the introduction of new technologies. In particular, medical technologies have provided expectant parents with videos and pictures they can use to introduce their unborn child to others before the birth. Internet technologies have further shaped the transition to parenthood by providing means through which expectant and new parents can share information about their experiences and their unborn child or newborn with family, friends, and acquaintances. According to McDaniel et al, “new mothers appear to be immersed in new age media, such as blogging and social networking,” yet, “research on mothers’ media use is still in its infancy” [1].

The Pew Research Center began tracking the use of social media sites in 2005 [2]. At that time, 7% of the American population reported using social media. Just 10 years later, 60% of Americans reported they were users of social media sites [2]. Recent statistics indicate there are over 1.5 billion Facebook users [3], with parents comprising a significant portion of social media users in the United States. Of the various social media sites in existence, Facebook is the most common platform used by parents in the United States. In particular, Duggan et al [4] found that 74% of parents reported using Facebook, and Hicks and Brown [5] found that 85% of the pregnant women in their study checked Facebook at least once every day. Of the Facebook-using parents in the Duggan et al [4] study, 75% of parents reported that they logged on to Facebook daily, whereas only 12% reported weekly or less than weekly use of the site. Similarly, Bartholomew et al [6] found that daily Facebook use was common among new mothers. Additional research focusing on the transition to parenthood also shows that new parents use social media on a weekly basis [1].

The Internet, and subsequently social media, has changed the way in which we search for and gather information. In fact, according to Daniels and Wedler, “information seeking through the Internet has become one of the easiest ways to learn about health-related information” [7]. Given the multitude of health-related issues that arise during parenthood, it is not surprising that parents rely on the Internet to gather health-related, as well as parenting information [8]. Pregnant women are no exception when it comes to using the Internet for informational purposes. Research indicates that pregnant women use the Internet to gain reassurance about the normalcy of their pregnancy and symptoms [9] and to gather information about pregnancy, birth, and labor [10,11]. According to Asiodu and colleagues, “social media platforms appeared to be the preferred mechanism for obtaining important information during the antepartum and postpartum periods” for the first-time mothers interviewed in their study [12], with the practice declining during the postpartum period.

Social media also serves a supportive function for expectant and new parents. Duggan et al [4] found that 42% of parents in their study received emotional or social support related to their parenting role through social media. Such support could be gained through online interactions and exchanges with friends and family on social media sites [13] and through membership in social media groups (ie, groups for new mothers). In particular, Tomfohrde and Reinke [8] found that social media fulfilled a supportive function for breastfeeding mothers, whereas new parents in the Thoren et al [14] study reported receiving support through an online support group for premature infants. The tendency of parents to share parenting challenges on Facebook is supported by the finding that social media users were more likely than those who did not use the social media sites to be “aware of stressful events in the lives of their close friends and more distant acquaintances” [15].

Finally, social media appears to facilitate bonding and connections for expectant and new parents [6]. Specifically, Lupton [10] reported that social media provided pregnant women with a sense of connection with their peers, as well as with their own unborn child. The practice of posting status updates and photos on social media appears to play a role in establishing and maintaining connections with others. Researchers have found that new parents shared images of their children on social media [1,10], with over two or three of the new parents in the Bartholomew et al [6] study posting photos of their children each month. In fact, parents in one study reported that posting pictures and comments about their children was their most common social media activity [16]. Prenatal ultrasound technology provides images, “baby’s first picture” [17], that can be shared prenatally by expectant parents, and research indicates that pregnant women share the ultrasound images to facilitate bonding and to involve others in their pregnancy [18]. Saetnan identified ultrasound technology as a “family-building technology,” noting its ability to involve others in the pregnancy, foster supportive interactions, and initiate “thinking about the baby as a family member” before birth [19]. Facebook and other social media platforms provide additional opportunities for expectant parents to share the technologically produced ultrasound images for these purposes, thus augmenting the notion of “family-building technology” to include social media. In fact, Johnson suggested that “Facebook may be one of the social communities in which women and their partners first announce their pregnancy and where they share ultrasound images, their experiences during pregnancy as well as their excitement at the impending arrival of their baby” [20].

Parental use of social media sites appears to vary by pregnancy status, gender, and age of the parent. In particular, women pregnant for the first time are more likely to use social media than multiparous women [21]_._ As is the case with social media use in general [2], younger parents (younger than 40 years) are more likely to use social media [22] and Facebook [4] than older parents. There are also gender differences in terms of which social media site is being used by parents, with mothers using Facebook significantly more than fathers for support (80% mothers, 65% fathers) and informational purposes (83% mothers, 74% fathers) [4]. Recently, Bartholomew et al [6] explored Facebook use during the postpartum period in relation to the parenting role, finding that new mothers were more likely than new fathers to utilize the social networking site.

### Prenatal Attachment

As the earliest conceptualization, the definition of prenatal attachment has evolved from encompassing maternal behaviors indicative of the mother’s affiliation toward and interaction with her unborn child [23], to later becoming a multidimensional construct that also involves expectant parents’ thoughts and fantasies about the fetus [24]. Doan and Zimmerman [25,26] further conceptualized prenatal attachment as involving multiple components—behavioral, cognitive, and affective. More recently, Krisjanous et al defined prenatal attachment as “the emotional attachment made up of feelings of afﬁliation and affection by the mother to the developing baby, which indicates positive acceptance and acknowledgment that the pregnancy is producing a person in their own right” [27].

Most relevant to this study, Condon and Corkindale [28] conceptualized prenatal maternal attachment as “a desire for knowledge about the fetus, pleasure in interaction with the fetus (both in fantasy and reality), and a desire to protect the unborn baby and his/her own needs, even at the expense of the mother’s own” [28]. More specifically, they conceptualized it as comprising two components—the quality of the pregnant woman’s attachment to and her preoccupation with her unborn child. The quality of prenatal attachment involves the pregnant woman’s positive feelings for and clear images of her unborn child, whereas the preoccupation component involves the strength of the pregnant woman’s attachment to her unborn child. According to Condon and Corkindale [28], the strength of her attachment is manifested through such processes as time spent thinking and talking about the unborn child and attempts to engage in behaviors that are healthy and protective of her unborn child.

Despite the various conceptualizations of the construct, research consistently indicates that prenatal attachment increases over the course of the pregnancy [29,30], with moderate stability carrying into toddlerhood [31]. Multiple factors have been investigated in relation to prenatal attachment, with research indicating that prenatal attachment is higher among women who are pregnant for the first time [32] and those who are involved in a supportive couple relationship [33,34]. In addition, certainty about fetal sex [29], quickening, and prenatal technology [35-38] have also been associated with maternal prenatal attachment.

Although parent-child attachment, during the postpartum period and later, typically receives more attention from researchers and interventionists, research has highlighted the importance of attachment during the prenatal period. In particular, researchers [33] found a positive association between maternal-fetal attachment and engagement in self-care behaviors and positive health practices during pregnancy. Furthermore, prenatal attachment also appears to be associated with cigarette smoking, a behavior that poses serious risks to the pregnancy and newborn [39]. Specifically, researchers found a negative correlation between maternal prenatal attachment and the number of cigarettes smoked by the woman during the pregnancy [40], as well as higher presence of particular elements of prenatal attachment among women who quit smoking during pregnancy versus those who did not [41]. Similarly, Ross [42] found a negative correlation between prenatal attachment and alcohol consumption during pregnancy. Overall, the research indicates that prenatal attachment is negatively associated with harmful maternal behaviors and positively associated with behaviors that are more optimal during pregnancy. It is then not surprising that prenatal attachment has also been associated with negative neonatal outcomes such as low birth weight [33,43].

### This Study

Although the associations between prenatal attachment and maternal behaviors during pregnancy have been investigated, the role of prenatal attachment in the mother’s representation of her unborn child to others has not been investigated. Furthermore, despite the importance of Facebook in parents’ lives, research on expectant parents’ use of Facebook to share pregnancy-related information is lacking. More specifically, to date, researchers have not investigated a potential relationship between prenatal maternal attachment and pregnancy-related posts on social media. On the basis of the findings of previous research and the components of attachment, as identified by Condon and Corkindale [28], it seems plausible that a relationship does exist between prenatal attachment and social media posts about one’s pregnancy and unborn child. In terms of the strength (preoccupation) component of prenatal attachment, one may assume that pregnant women who are more attached to their unborn child would post about their pregnancy and unborn child more frequently to Facebook than those who are less attached to their unborn child, as they are spending more time thinking about their unborn child. In addition, the avoidance of harmful behaviors, an additional aspect of the strength component of prenatal attachment, may embolden a pregnant woman to seek advice and support regarding her pregnancy and unborn child’s health from others on social media. Finally, a pregnant woman who has clearer images of and more positive feelings about her unborn child, both indicators of the quality of prenatal attachment, would likely be more compelled to post about her pregnancy and unborn child in attempts to involve others in her pregnancy (to share in her excitement) and to introduce her unborn child as a member of the family. Taken together, therefore, it is hypothesized that pregnant women utilize social media, specifically Facebook, to share pregnancy-related information with others and that such sharing is related to the pregnant woman’s prenatal attachment toward her unborn child.

Specifically, the study examined the use of Facebook to share information about pregnancy among pregnant women, as well as any association between prenatal attachment and sharing pregnancy-related information on Facebook.

The following research questions (RQs) were explored in the study:

- RQ1. What pregnancy-related information are pregnant women sharing on Facebook?
- RQ2. With whom are they sharing pregnancy-related information on Facebook?
- RQ3. How often do they share pregnancy-related information on Facebook?
- RQ4. Why do they share pregnancy-related information on Facebook?

In addition, the following hypotheses (Hs) were tested in the study:

- H1. Prenatal attachment will be associated with frequency of sharing pregnancy-related information on Facebook.
- H2. Prenatal attachment will be associated with how many types of pregnancy-related information are shared on Facebook
- H3. Prenatal attachment will be associated with the number of groups with whom pregnancy-related information is shared on Facebook
- H4. Prenatal attachment will be associated with the reasons for sharing pregnancy-related information on Facebook.

## Methods

### Recruitment

Upon receiving Human Subject Approval, pregnant women who were at least 18 years of age were recruited for participation in the study through posts to the researcher’s personal and research Facebook pages that were subsequently shared by others, paid advertisements on Facebook that were targeted to pregnancy-related groups and pages, and posts to listservs of the researcher’s national professional organization. Individuals interested in participating were directed through the announcement to the secure Web-based survey system utilized by the university where they completed the consent form and the survey. In addition to the consent form, the survey consisted of one page of demographic questions, one page of questions assessing the use of Facebook to share pregnancy-related information, and one page of questions comprising the attachment scale for a total of 41 questions. To facilitate completion and expedite movement through the survey, question condition settings were included to automatically skip questions that were not relevant to participants. Due to university human subject stipulations, participants were allowed to exit the survey at any time and skip any questions in the survey with the exception of indicating their consent, or lack thereof, to participate on the consent form. Participants were instructed to complete the survey with their current pregnancy as the focus of their answers. The survey was open and set for single response submission, without the capability to update responses after submission; although, participants were able to return to previous questions before submission.

Before data collection, the Web-based survey was pilot tested by four women known to the researcher—two of whom were pregnant and two who had recently given birth. Issues with survey formatting and word choice were resolved based on feedback provided from the pilot participants. In addition, the pilot participants provided the researcher with information concerning the time required for survey completion, which was subsequently used for the time-to-complete estimate provided on the consent form.

### Measures

#### Dependent Variables

Frequency of sharing information, types of information shared, with whom information was shared, and reasons for sharing information were the dependent variables in this study. Each was measured with closed-ended questions on the survey.

##### Frequency of Sharing Information

Participants indicated how often they posted pregnancy-related information to Facebook during the current pregnancy by choosing one of the following options: (1) less than once per month, (2) once per month, (3) a few times per month, (4) once per week, (5) a few times per week, (6) once per day, or (7) more than once per day. The options were developed by the researcher using categories from the Bartholomew et al [6] study as a framework. A higher number represented more frequent sharing of pregnancy-related information on Facebook.

##### Types of Information Shared

Participants indicated which of the following types of pregnancy-related information they had shared on Facebook during their current pregnancy by indicating “yes” or “no” for each type: (1) announcement of their pregnancy, (2) ultrasound pictures or videos of their unborn child, (3) announcement of their unborn child’s sex, (4) information about their pregnancy symptoms, (5) information about their preparation for the baby, (6) information about medical appointments, (7) information about the progression of the pregnancy, (8) information about pregnancy complications, and (9) information about the birth plans. These categories were developed by the researcher based on the researcher’s previous research with expectant parents and personal correspondence with pregnant women about the types of pregnancy-related information they shared with others. Each item was coded, with a 1 indicating the participant shared the information and 0 indicating the participant did not share the information on Facebook. Scores on the nine items were summed, with a higher score indicating the sharing of more types of pregnancy-related information on Facebook.

##### Whom Shared With

Participants indicated which of the following groups they shared pregnancy-related information with on Facebook: (1) only family, (2) only friends, (3) only friends and family, or (4) public (no restrictions on who could see the information). These categories were developed by the researcher, with the privacy settings available to Facebook users (public, friends, and customize) serving as the initial framework for the categories. A “yes” response to each category was coded as 1 and a “no” response coded as 0. Scores for the four groups were summed to gain a *whom shared with* score (ranging from 0-4), with a higher number indicating more groups with whom the information was shared.

##### Reasons for Sharing

Participants indicated the reasons they posted pregnancy-related information to Facebook by indicating which of the following options applied to them: (1) to share excitement, (2) to document pregnancy, (3) to get advice, (4) to involve others in the pregnancy, (5) to issue a prayer request, and (6) others expected them to share it. The response options were developed by the researcher, utilizing reasons cited in literature and from personal correspondence with pregnant women. A response of “yes” for an item was coded as 1, whereas a “no” response was coded as 0. Subsequent factor analysis was performed on the six reasons for sharing information, with the following four categories of reasons identified: (1) getting advice, (2) issue prayer request, (3) involve others or share experience, and (4) expected to.

#### Independent Variables

Prenatal attachment served as the independent variable in the study. In addition, demographic variables were treated as controls in the analyses.

##### Prenatal Attachment

Prenatal attachment was assessed with the 19-item Maternal Antenatal Attachment Scale (MAAS) [28,44]. The MAAS consists of two subscales. The first subscale, which is comprised of 10 items, assesses the pregnant woman’s *quality* of attachment to the unborn child (ie, her positive feelings about the unborn child and clear mental images of the unborn child), whereas the second 8 item *preoccupation* subscale assesses the strength of the pregnant woman’s attachment with her unborn baby (eg, the amount of time she thought about the unborn baby and protective behaviors). Examples of questions from the quality subscale are “Over the past two weeks when I think about the baby inside me I get feelings which are (very sad, moderately sad, a mixture of happiness and sadness, moderately happy, very happy)” and “The picture in my mind of what the baby at this stage actually looks like inside the womb is (very clear, fairly clear, fairly vague, very vague, I have no idea at all).” Items from the preoccupation subscale include “Over the past two weeks I have had dreams about the pregnancy or baby (not at all, occasionally, frequently, very frequently, almost every night)” and “Over the past two weeks I have taken care with what I eat to make sure the baby gets a good diet (not at all, once or twice when I ate, occasionally when I ate, quite often when I ate, every time I ate).” Per the instrument guidelines, one question (“Over the past two weeks I have felt that the baby inside me is dependent on me for its well-being”) was included in the overall attachment score, but not in either of the subscales. Participants indicated their level of agreement with each statement on a 5-point Likert scale, with a total computed for the full scale and each subscale, and higher totals indicating greater prenatal attachment.

##### Control Variables

Participants provided the following information: (1) age (5 categories), (2) parity status (first pregnancy vs not first pregnancy), (3) knowledge of fetal sex (yes or no), (4) weeks currently pregnant (6 categories), and (5) planned pregnancy (yes, no). Specific categories for the control variables that appeared on the survey are listed in Table 1.

### Statistical Analysis

Statistical Package for the Social Sciences (SPSS) version 23 (IBM Corp) was used for all analyses. Descriptive statistics were used to analyze the data for research questions 1 to 4. Correlations (Pearson, Spearman rho) were used to test hypotheses 1 to 4, with multiple and binomial logistic regression used to test for associations between the independent and dependent variables when controlling for the other variables and demographic variables (participant’s age, first pregnancy status, knowledge of fetal sex, number of weeks pregnant, and planning of the pregnancy). Only the attachment subscales (preoccupation and quality), and not the overall prenatal attachment scores, were included in the regression analyses to reduce multicollinearity between the overall scale and subscale scores.

## Results

### Participant Characteristics

There were 5395 clicks on the survey link during the 7-month data collection period. From those clicks, 218 individuals consented to participate in the study, and a total of 117 pregnant women completed the Web-based survey. The sample was predominately white (90/109, 82.6%), married (85/110, 77.3%), college educated (74/113, 65.5%), and in the age range of 26 to 29 years (42/114, 36.8%). In addition, almost half of the women were pregnant for the first time (56/113, 49.6%), most of the pregnancies were planned (76/113, 67.3%), and the most common category for weeks pregnant was 27 to 33 weeks (46/113, 40.7%). See Table 1.

The mean on the overall attachment scale was 75.68 (SD 7.655; range: 49-88.70). The mean on the preoccupation (strength) subscale was 28.66 (SD 4.967; range: 15-38.70), and the mean for the quality subscale was 42.38 (SD 3.569; range: 20.41-47).

### Research Questions

#### Research Question 1

The most common type of information shared on Facebook was the pregnancy announcement (75/108, 69.4%), followed by sharing pregnancy progress (57/110, 51.8%), and announcing the fetus’ sex (55/111, 49.5%). The least common type of information shared was birth plans (7/116, 6.0%). See Table 2 for more complete results.

#### Research Question 2

Friends and family were the most common recipients of the pregnancy-related information posted to Facebook. Over 75% of the women shared information with friends (91/116, 78.4%) and family (90/116, 77.6%). The women were less likely to share with individuals they did not know personally (Table 2).

#### Research Question 3

The majority of the participants posted pregnancy-related information relatively infrequently, with 44.4% (52/117) indicating they posted information related to their current pregnancy less than once per month, followed by sharing a few times per month (18.8%, 22/117). A smaller percentage of women shared information a few times per week, with even fewer sharing pregnancy-related information on a daily basis (Table 2).

#### Research Question 4

When considering all six of the reasons for sharing, the most common reason was to share the excitement of the pregnancy with others (57.9%, 62/107), followed by the desire to document the pregnancy (31.3%, 35/112), and get advice (28.9%, 33/114). The least common reason for sharing was feeling pressured by others to share the information (Table 2), indicating that most of the women voluntarily shared the information with others.

**Table 1:** Demographic information.

Demographic characteristics		n (%)
**Age, years**		
	18-21	12 (10.7)
	22-25	19 (16.7)
	26-29	42 (36.8)
	30-33	29 (25.4)
	34-39	9 (7.9)
	40-44	1 (0.9)
	≥45	0 (0.0)
**Race**		
	White	90 (82.6)
	Black	8 (7.3)
	Hispanic or Latino	4 (3.7)
	Native American	1 (0.9)
	Asian or Pacific Islander	5 (4.6)
	Other	1 (0.9)
**Annual income (USD)**		
	<30,000	25 (22.7)
	30,000-49,999	17 (15.5)
	50,000-74,999	20 (18.2)
	75,000-99,999	18 (16.4)
	>100,000	30 (27.2)
**Education**		
	Less than high school	3 (2.7)
	High school or general equivalency diploma	14 (12.4)
	Some college or vocational training	22 (19.5)
	Associate degree	3 (2.7)
	Bachelor’s degree	37 (32.7)
	Master’s degree	23 (20.4)
	Doctorate degree	11 (9.7)
**Marital status**		
	Single, never married	19 (17.3)
	Divorced	3 (2.7)
	Separated	2 (1.8)
	Widowed	1 (0.9)
	Married	85 (77.3)
**Weeks pregnant**		
	Under 13 weeks	5 (4.4)
	13 to 19 weeks	16 (14.2)
	20 to 26 weeks	16 (14.2)
	27 to 33 weeks	46 (40.7)
	34 to 40 weeks	29 (25.7)
	Over 40 weeks	1 (0.9)

**Table 2:** Sharing pregnancy-related information.

Category of sharing behavior		n (%)
**Type of information shared**		
	Pregnancy announcement	75 (69.4)
	Pregnancy progress	57 (51.8)
	Sex of fetus	55 (49.5)
	Ultrasound pictures	46 (39.3)
	Pregnancy symptoms	36 (32.1)
	Preparation for baby	33 (29.2)
	Medical appointments	17 (15.0)
	Pregnancy complications	14 (12.2)
	Birth plans	7 (6.0)
**With whom shared**		
	Friends	91 (78.4)
	Family	90 (77.6)
	Friends of friends	15 (12.9)
	Everyone	7 (6.0)
**Frequency shared**		
	Less than monthly	52 (44.4)
	Once per month	11 (9.4)
	Few times per month	22 (18.8)
	Once per week	8 (6.8)
	Few times per week	16 (13.7)
	Once per day	6 (5.1)
	More than once each day	2 (1.7)
**Reasons for sharing**		
	Share excitement	62 (57.9)
	Document pregnancy	35 (31.3)
	Get advice	33 (28.9)
	Involve others	19 (17.0)
	Issue prayer request	18 (15.9)
	Others expected it	9 (7.8)

### Hypotheses

#### Hypothesis 1

Results of Pearson’s correlations indicated that overall prenatal attachment and the quality and preoccupation subscales were positively and significantly associated with *frequency of sharing* (Table 3). Participants who reported higher overall prenatal attachment were more preoccupied with their unborn child and had more positive feelings about their unborn child shared information about their current pregnancy more frequently on Facebook.

Using the enter method of multiple regression, the control variables and quality and preoccupation subscale scores were entered in the equation. Results indicated that the age of participants was found to be a significant predictor, and the preoccupation subscale retained significance after controlling for other variables (Table 4). However, the quality subscale of attachment was no longer a significant predictor of frequency of sharing information. Participants who were younger and more preoccupied with their unborn baby shared pregnancy-related information more frequently on Facebook. Therefore, H1 was partially supported as the preoccupation subscale of attachment was found to be significantly associated with frequency of sharing pregnancy-related information on Facebook after controlling for other variables.

#### Hypothesis 2

Overall, prenatal attachment and the two subscales (preoccupation and quality) were significantly associated with sharing *more types* of pregnancy-related information on Facebook (Table 3). Specifically, participants who reported higher overall prenatal attachment, higher quality attachment, and more preoccupation with their unborn child shared more types of pregnancy-related information on Facebook.

The enter method of multiple regression was used to test the associations after controlling for other variables. Results indicated that the preoccupation subscale, age, and weeks pregnant were significant contributors to the model, with those who were more preoccupied with their unborn child, younger, and further along in their pregnancies sharing more types of information (Table 5). The quality of attachment was no longer significant after controlling for the influence of other variables. Therefore, H2 was partially supported with the preoccupation subscale of attachment being significantly and positively related to the total types of pregnancy-related information shared on Facebook.

#### Hypothesis 3

Analysis with Pearson correlation revealed that overall prenatal attachment and the preoccupation subscale were significantly associated with sharing pregnancy-related information with *more groups* on Facebook (Table 3). Specifically, participants who reported higher overall prenatal attachment and were more preoccupied with their unborn baby shared pregnancy-related information with more groups of people on Facebook.

The enter method of multiple regression model was again used to test associations between prenatal attachment and number of groups with whom information was shared while controlling for other variables. Age and preoccupation with the unborn baby were significant contributors to the model (Table 6). H3 was partially supported in that participants who were more preoccupied with their unborn baby (a subscale of attachment) shared pregnancy-related information with significantly more groups of people on Facebook.

**Table 3:** Correlations between independent and dependent variables.

Variables	Prenatal attachment	Preoccupation attachment subscale	Quality attachment subscale
Frequency shared	.338^a^	.308^a^	.228^a^
Total types shared	.332^a^	.360^a^	.194^b^
Groups shared with	.248^a^	.338^a^	.050
Share pregnancy	.338^a^	.274^a^	.303^a^
Get advice	.236^b^	.277^a^	.091
Prayer request	.251^a^	.238^a^	.163
Expected to	−.135	−.096	−.177

^a^Correlation significant at the .01 level.

^b^Correlation significant at the .05 level.

**Table 4:** Multiple regression results for hypothesis 1 (frequency of sharing; N=108; *R*^2^=.227, *F*_8,100_=3.671, and *P*<.001).

Variable		B (SE)	Beta	*t* value^a^	*P* value
Constant		1.349 (2.366)	N/A	0.570	.57
**Controls**					
	Age	−.488 (0.149)	−.336	−3.286	.001
	Number of children	.358 (0.230)	.194	1.556	.12
	Fetal sex	−.428 (0.433)	−.105	−0.98	.32
	Weeks pregnant	−.050 (0.079)	−.065	−0.625	.53
	Planned pregnancy	.012 (0.350)	.003	0.035	.97
	First pregnancy	.438 (0.416)	.131	1.055	.29
**Predictors**					
	Preoccupation (attachment)	.090 (0.035)	.262	2.554	.01
	Quality (attachment)	−.010 (0.054)	−.021	−0.185	.85

^a^Degrees of freedom for *t* test values=8,100.

**Table 5:** Multiple regression results hypothesis 2 (number of types of information shared; N=105; *R*^2^=.314, *F*_8,97_=5.540, *P*<.001).

Variable		B (SE)	Beta	*t* value^a^	*P* value
Constant		−2.815 (3.308)	N/A	−.851	.4
**Controls**					
	Age	−.717 (0.208)	−.337	−3.454	.001
	Number of children	.474 (0.322)	.174	1.471	.14
	Fetal sex	−.735 (0.610)	−.121	−1.206	.23
	Weeks pregnant	.328 (0.111)	.291	2.940	.004
	Planned pregnancy	.378 (0.500)	.072	0.756	.45
	First pregnancy	.552 (0.581)	.112	0.949	.35
**Predictors**					
	Preoccupation (attachment)	.129 (0.050)	.254	2.605	.01
	Quality (attachment)	.038 (0.076)	.053	0.498	.62

^a^Degrees of freedom for *t* test values=8,97.

**Table 6:** Multiple regression hypothesis 3 (groups with whom information shared; N=107; *R*^2^=.252, *F*_8,99_=4.159, *P*<.001).

Variable		B (SE)	Beta	*t* value^a^	*P* value
Constant		1.570 (2.344)	N/A	0.670	.51
**Controls**					
	Age	−.348 (0.150)	−.233	−2.313	.02
	Number of children	.137 (0.234)	.072	0.585	.56
	Fetal sex	−.127 (0.442)	−.030	−0.287	.77
	Weeks pregnant	−.052 (0.080)	−.066	−0.641	.52
	Planned pregnancy	−.586 (0.355)	−.161	−1.649	.10
	First pregnancy	.175 (0.421)	-.051	-.415	.68
**Predictors**					
	Preoccupation (attachment)	.125 (0.036)	.350	3.467	.001
	Quality (attachment)	−.016 (0.055)	−.032	−0.285	.77

^a^Degrees of freedom for *t* test values=8,99.

#### Hypothesis 4

Pearson correlation results indicated that overall prenatal attachment and the preoccupation and quality of attachment subscales were significantly and positively associated with sharing information to *involve others or share the pregnancy experience* (Table 3) *.* Results of the multiple regression analysis indicated that after controlling for the other variables, age, weeks pregnant, and quality of attachment were significantly associated with sharing to involve others (Table 7). The preoccupation subscale was no longer significant after controlling for the other variables. Participants who were younger, further along in their pregnancy, and reported higher quality of attachment were significantly more likely to share pregnancy-related information to involve others or share the pregnancy experience with others.

Spearman rho correlational analysis indicated that, overall, prenatal attachment and the preoccupation attachment subscale were significantly and positively associated with sharing to *get advice* from others (Table 3). Binomial logistic regression analysis indicated that after controlling for other variables, the preoccupation attachment subscale retained significance (Table 8), meaning that individuals who reported greater preoccupation with their unborn baby were significantly more likely to post pregnancy-related information to Facebook in order to get advice from others.

Results of Spearman rho analysis showed that overall prenatal attachment and the preoccupation attachment subscale were significantly, positively associated with sharing pregnancy-related information to *issue a prayer request* (Table 3). However, after controlling for other variables using binomial logistic regression, the model was not significant, χ^2^_8_=3.5, N=108, *P*=.90, nor were the independent or control variables. Thus, prenatal attachment was not significantly associated with sharing to issue a prayer request after controlling for other variables.

**Table 7:** Multiple regression results hypothesis 4 (sharing to involve or share experience; N=101; *R*^2^=.229, F_8,93_=3.460, *P*=.002).

Variable		B (SE)	Beta	*t* value^a^	*P* value
Constant		−4.427 (1.998)	N/A	−2.216	.03
**Controls**					
	Age	−.319 (0.126)	−.266	−2.533	.01
	Number of children	.172 (0.192)	.114	0.896	.37
	Fetal sex	−.037 (0.372)	−.011	−0.099	.92
	Weeks pregnant	.146 (0.070)	.229	2.084	.04
	Planned pregnancy	−.097 (0.297)	−.327	−0.327	.74
	First pregnancy	.049 (0.350)	.139	0.139	.89
**Predictors**					
	Preoccupation (attachment)	.034 (0.030)	.119	1.132	.26
	Quality (attachment)	.121 (0.046)	.304	2.654	.009

^a^Degrees of freedom for *t* test values=8,93.

**Table 8:** Logistic regression results for hypothesis 4 (sharing to get advice; N=108; χ^2^_8_=7.5, *P*=.48).

Variable		B (SE)	Wald chi-square^a^	*P* value
Constant		−2.439 (3.595)	0.5	.497
**Controls**				
	Age	−.325 (0.237)	1.9	.17
	Number of children	−.135 (0.376)	0.1	.72
	Fetal sex	−.404 (0.666)	0.4	.54
	Weeks pregnant	.197 (0.130)	2.3	.13
	Planned pregnancy	.504 (0.539)	0.9	.35
	First pregnancy	.359 (0.654)	0.3	.497
**Predictors**				
	Preoccupation (attachment)	.138 (0.060)	5.3	.02
	Quality (attachment)	−.056 (0.087)	0.4	.52

^a^Degrees of freedom for Wald chi-square=8.

Finally, results of Spearman rho analysis indicated that none of the attachment scores were significantly associated with sharing because of the *expectations of others*. Therefore, the overall prenatal attachment score and the quality and preoccupation subscales were not significantly associated with sharing pregnancy-related information on Facebook to conform to others’ expectations to do so.

## Discussion

### Principal Findings and Comparison With Prior Work

The women who participated in the study reported sharing pregnancy-related information relatively infrequently on Facebook. In fact, the majority of the women reported sharing the information less than monthly, with less than 7% of the women sharing information on a daily basis. This finding contradicts that of previous research that indicated 75% of parents [4] and 85% of pregnant women [5] checked Facebook on a daily basis. However, this discrepancy in results may be because of the fact that this study did not explore the frequency of general Facebook use (ie, using Facebook for purposes other than sharing pregnancy-related information). Instead, the participants were asked only how frequently they shared pregnancy-related information. Therefore, it may be the case that the participants did log on to Facebook more frequently for general use than they did to post pregnancy-related information. Furthermore, it is likely that pregnancy-related information is shared on Facebook relatively infrequently because the salience of the information is infrequent. When there is something novel to post, pregnant women post it; however, such novel information is likely to be a one-time occurrence or relatively infrequent. In support of this explanation, two of the three most commonly shared types of information by women in this study reflected one-time occurrences (announcing the pregnancy and sharing the fetus’ sex).

The findings of this research also showed that the women voluntarily shared pregnancy-related information with a rather select group of individuals, namely their friends and family on Facebook. Overall, very few shared because of the expectations of others, and few shared with individuals they did not know (ie, friends of friends and everyone on Facebook). The most common reasons for sharing the information fit the overall category of sharing to involve others or share the excitement of the pregnancy. By sharing information about their pregnancy, unborn child, and preparations, they were providing others access to their pregnancy experience and enhancing connections with others during their pregnancy in their role as an expectant mother. Therefore, the findings appear to support researcher assertions that social media use facilitates connection for pregnant women [6,10], and Facebook specifically may provide new parents opportunities to maintain social ties during the transition to parenthood [6].

Aside from the aforementioned reasons, posting to gain advice from others was the third most common reason for sharing pregnancy-related information on Facebook, with one-third of the sample reporting this reason. This finding corresponds to previous research documenting the value of social media as an information-gathering tool [7] and the practice of pregnant women seeking information and advice through social media [9-11]. Unfortunately, the actual content of the women’s posts to Facebook was not investigated in this study, thus leaving the specific types of advice they were seeking unknown. However, given that pregnancy symptoms were shared by over one-third of the sample, it is possible that a portion of the advice they sought was related to the symptomology of pregnancy, as well as advice related to the other types of information shared by the women (ie, preparing for baby, complications, and birth plans).

The four types of pregnancy-related information most commonly shared on Facebook by the women in this study were the pregnancy announcement, information about the progression of the pregnancy, the sex of their unborn child, and ultrasound photos. In general, these findings support Johnson’s [20] assertion that Facebook provides expectant parents with opportunities to announce their pregnancy and share images of their unborn baby with others. In terms of specific findings, 69% of the pregnant women in this study reported they had announced their pregnancy via Facebook, whereas 39% reported they had shared an ultrasound image of their unborn child on Facebook. The latter finding coincides with previous research in which sharing comments and photos of one’s child or children was the most common activity carried out by parents on Facebook [16]. Although the percentage of pregnant women who shared an ultrasound image in this study is lower than that reported among new parents sharing photos in the Bartholomew et al [6] study, the difference may be because of a lack of access to a quality ultrasound image, whereas new parents typically accrue many photos of their newborn that are suitable and available for sharing. Given that the study participants did not report on their possession of ultrasound images, this explanation is merely speculative. However, the Bartholomew et al [6] finding that new parents reported uploading and posting more pictures of their child during the postpartum period than they did during the pregnancy lends support to this explanation.

In general, the findings regarding sharing pregnancy-related information on Facebook provide support for the family-building capabilities of technology. Although past research indicated that prenatal technology assisted with introducing an unborn child as a member of the family and building an identity for the unborn child in the family [17,19], our findings indicate that social media also serves as a family-building tool during the prenatal period. The pregnant women in this study voluntarily shared information about their pregnancy and preparations with others to facilitate involvement in the pregnancy experience and, most likely, to establish connections. In addition, the women shared specific information about their unborn child on Facebook, specifically the unborn child’s sex and ultrasound image. Thus, by way of sharing information, their unborn child could establish an identity before birth, as previously suggested by Johnson [20], among those who viewed the posts. In particular, the image could allow others to explore the child’s physical features for family resemblance, while information about the unborn child’s name, prenatal habits (ie, kicks), and sex could facilitate the development of the child’s role within the family [19] and speculations about his or her future personality, interests, and behaviors.

This is the first study to investigate the role of prenatal attachment with respect to pregnant women sharing information about their pregnancy on Facebook, with results indicating that prenatal attachment is positively related to sharing pregnancy-related information. To begin, results of correlational analyses showed that prenatal attachment, in general, was positively and significantly related to all aspects of sharing pregnancy-related information, with the exception of sharing because of expectations. Thus, pregnant women who were more attached to their unborn child were more likely to voluntarily post information related to their pregnancy or unborn child on Facebook. More specifically, before controlling for other variables, the preoccupation component of prenatal attachment was significantly and positively associated with all aspects of sharing, again with the exception of sharing because of others’ expectations. This finding makes intuitive sense. One would expect that a pregnant woman who possesses a stronger attachment to her unborn child, meaning she spends more time thinking about and has stronger feelings for her unborn child, would post more pregnancy-related information on Facebook than a pregnant woman who spends less time preoccupied with her unborn child.

These findings may also be interpreted in relation to the concept of maternal identity or, more specifically, maternal identify confirmation. According to Allen and Hawkins, maternal identity confirmation is the “desire for the external validation of the maternal role” [45]. Recently, this concept was investigated in relation to Facebook use among parents. Specifically, Schoppe et al [46] found positive relationships between maternal identity confirmation and aspects of Facebook activity. In particular, women who sought more confirmation of their maternal identity were more likely to post photos of their child and to use their child’s photo as their own profile picture on Facebook. Drawing on Schoppe et al’s findings, it may be that salience of one’s maternal identity played a role in the sharing of pregnancy-related information on Facebook in this study. More specifically, pregnant women for whom the maternal identify was more salient may have posted more pregnancy-related information on Facebook to receive validation of their maternal role and affirmation “that they are doing motherhood (in this case, pregnancy) correctly and normatively” [46].

Overall, the findings of this study indicate that, when considering the two subscales of attachment used in this study, the *strength,* or preoccupation component, of prenatal attachment is associated with more aspects of sharing pregnancy-related information on Facebook than is the *quality* component of prenatal attachment. Before controlling for other variables in the regression analyses, the quality subscale was associated with three aspects of sharing, whereas the preoccupation subscale was associated with six aspects of sharing. More specifically, after controlling for other variables, quality of attachment, which involves the positive feelings the woman has about her unborn child, was significantly associated with only sharing to involve others or share the pregnancy. In contrast, the strength or preoccupation component of prenatal attachment was significantly associated with frequency of sharing, number of types of information shared, number of groups with whom information was shared, and sharing to get advice. The question arises, “Why is strength of prenatal attachment significantly related to these aspects of sharing when quality of the attachment is not?” Obviously, a causal influence cannot be determined by the methodology used in the study. However, the explanation may be as simple as women who are more preoccupied with their unborn child or pregnancy manifest that preoccupation by sharing more types of information about their pregnancy more frequently and with more people. In addition, their preoccupation may involve anxiety about the pregnancy or unborn child that, in turn, prompts them to seek advice, validation, or reassurance on Facebook. Research on adult attachment and Facebook activity may provide support for this explanation in that adults who are more anxious about their relationships (ie, anxiously attached) are more likely to post about and seek visibility of their romantic relationships on Facebook [47]. However, this evidence should be viewed with caution, given that adult attachment and prenatal attachment are different constructs, as the former focuses on the adult within romantic and intimate relationships [48], whereas the latter focuses on the adult’s thoughts, feelings, and behaviors toward or about their unborn child and hence not within the context of a bidirectional relationship. That said, although one may argue that the applicability of adult attachment research to this study is questionable, it does shed some light on the potential role of anxiety in posting of pregnancy-related information on Facebook, particularly in relation to the strength or preoccupation component of prenatal attachment.

Similarly, it may also be that the pregnant women who were more preoccupied with their unborn child possess a personality trait, or other characteristic, that played a role in their sharing behavior, as well as their tendency to be more preoccupied with their unborn child. Neuroticism, which involves anxiety and worry, is one such trait that may moderate the relationship between prenatal attachment and sharing of pregnancy-related information on Facebook. Although research on personality types and social media use during pregnancy is lacking, there is evidence to suggest that the personality trait of neuroticism is positively related to Facebook activity among adults [49,50]. With respect to parenting, although Schoppe-Sullivan et al [46] did not find significant relationships between personality types and frequency of Facebook activity among parents, they did find that parents who scored higher on neuroticism posted pictures of their child sooner after the child’s birth. Therefore, it could be that pregnant women who are also neurotic are more preoccupied about their unborn child and, in turn, use Facebook as a platform to solicit information, reassurance, and support. Future research is needed to refute or support this potential moderating effect.

Though not a specific focus of this research, findings related to the control variables are worthy of mention. Specifically, in the regression analyses, weeks pregnant and age were significantly associated with aspects of sharing pregnancy-related information on Facebook. Age was negatively and significantly associated with frequency of sharing, number of types of information shared, number of groups with whom information was shared, and sharing to involve others. These findings confirm prior research that indicated a negative correlation between Facebook use and age among the general population and parents [4,2]. Furthermore, weeks pregnant, another control variable in this study, was found to have a significant positive association with aspects of sharing pregnancy-related information on Facebook. Participants who were further along in their pregnancy shared more types of information and were more likely to share to involve others in the pregnancy. This finding is not surprising given that, as the pregnancy progresses, there are more opportunities and more types of pregnancy-related information to share (ie, more frequent appointments, preparations for birth, etc), and prenatal attachment has been shown to increase throughout gestation [29,30]. Another worthy explanation may involve Rothman’s [51] notion of the “tentative pregnancy” in which women suspended attachment and excitement about their pregnancy until they were assured of the viability and health of the fetus through prenatal testing. In particular, and of relevance to this study, the women interviewed by Rothman postponed announcing their pregnancy until they received the results of prenatal testing that confirmed the fetus’ health or their continuation of their pregnancy. More recently, Ross [52] asserted that the concept of “tentative pregnancy” could be extended to pregnancy in general, not only to instances of genetic testing. Thus, the women in this study may have delayed or suspended posting about their pregnancy until they were more assured of the health and viability of their pregnancy and unborn child. Given that our society perceives the first trimester as a period of risk and many discourage announcing a pregnancy during this period, it is worth noting the possibility that the “tentative pregnancy” may have played a role in the women’s posting behavior and, subsequently, the findings of this study. The women did not report *when* they posted about the pregnancy on Facebook; therefore, it is not possible to ascertain if this explanation is accurate or fitting of the women in this study. However, women in Ross’ research “engaged with the convention of keeping news of their pregnancy secret during its early stages” [52]. Therefore, it seems plausible that the women in this study succumbed to this practice as well.

### Limitations and Suggestions for Future Research

Despite its contributions to existing literature, this study was not without limitations. To begin, the sample size was relatively small and homogenous with regard to demographic characteristics. This may have impacted the strength of the relationships between the variables, while also limiting the generalizability of the findings. Paid advertisements were posted to groups on Facebook, with target recipients representing diverse demographics. Yet, the sample was predominately white, married, and college educated. The Facebook posts advertising the study likely played a role in the homogenous sample characteristics, as individuals may have subsequently shared the post with friends and family who possessed similar demographic characteristics. In addition, the pregnant women who participated in the study were also a self-selected group who may have possessed a stronger interest in or deterrence to Facebook than the average pregnant woman. Future research would benefit from utilizing additional recruitment methods that are more enticing and accessible to a wider diversity of individuals. In particular, face-to-face and nonsocial media–related recruitment may reach a more diverse group of women. In addition, overall participation and survey completion may have been increased, resulting in a larger sample size, if an incentive had been offered to participants who completed the survey.

Aside from sample characteristics, there were additional limitations to this study which are worthy of note. As previously discussed, the women were not asked *when* they shared the pregnancy-related information, nor were they asked *where* they shared the information. Given that recruitment advertisements were posted to group pages that focused on pregnancy, one can assume that some portion of the sample participated in Facebook groups related to pregnancy. Although the women did indicate who they shared the information with, the categories were not specific enough to ascertain if the information they shared with friends or everyone was shared on group pages or on their own personal Facebook page or if Facebook group members fit within their definition of friends for survey purposes. It would be interesting to investigate if there are differences in terms of frequency, content, and rationale for posts of pregnancy-related information to one’s own page versus those to group pages. Such information would be useful to professionals as they seek to incorporate social media within their services for pregnant women.

Finally, the timing of data collection may represent a limitation of the study. Participants were required to reflect upon their use of Facebook during their pregnancy. Recall error may have occurred when reporting how often, what types, and reasons for sharing pregnancy-related information on Facebook. In fact, Moore and McElroy encouraged researchers to use “actual Facebook data where possible and rely on survey data for information that cannot be obtained objectively” [53]. In addition to analyzing actual Facebook posts, an alternative method would involve requiring participants to record their daily use of Facebook for sharing pregnancy-related information through a Web-based portal or mobile app. These methods would overcome the challenge of participants accurately reflecting on their posting of pregnancy-related information on Facebook.

### Conclusions

The results of this study fill a gap in our knowledge about pregnant women’s use of Facebook to share information about their pregnancy, as well as the role of prenatal attachment in such sharing. The findings supplement previous research linking prenatal attachment to healthy behaviors and self-care by also showing a link between prenatal attachment and sharing information about one’s unborn child and pregnancy via social media. Although one certainly cannot endorse or encourage assessing prenatal attachment through Facebook posts, the results of this study are valuable in terms of the additional insight provided regarding associations between prenatal attachment and maternal behaviors.

Perhaps equally, or more, important to our existing knowledge are the descriptive findings of this study and their implications. The pregnant women in this study used Facebook to share pregnancy-related information for a variety of reasons, demonstrating the use of the social media platform during pregnancy for relational, supportive, and informational purposes. In particular, the findings support the suggestion of Bartholomew et al that “conceptions of new parents’ social support networks need to be expanded to include the online environment in addition to family, friends, and community members that new parents may see face to face” [6]. To maximize support networks, particularly for pregnant women who lack proximal support, professionals should be cognizant of the potential value of support garnered through social media. Finally, given that pregnant women in this study sought information and advice through Facebook, combined with the fact that an abundance of inaccurate information is available on the Internet and likely shared on social media, the current research further legitimizes a need for professionals to utilize Facebook and other social media platforms to dispense medically accurate information to pregnant women. In particular, support and information may be delivered by medical professionals through closed Facebook groups for patients to “join” [13], Facebook pages that patients “like” to receive medically accurate information and links to other credible sources [6], and Facebook Live sessions that allow pregnant women to interact with professionals in a “live question-and-answer online forum” [10]. Overall, the findings of this study lend merit to the use of Facebook by antenatal medical professionals and educators who are seeking alternative methods for providing information and fostering support among pregnant women via social media in our technology-driven society.

## Abbreviations

H: hypothesis
RQ: research question
